# Supplementary material for: Hydrogen bond donors and acceptors are generally depolarized in α‐helices as revealed by a molecular tailoring approach
Source: J Comput Chem. 2019 May 17;40(23):2043–52. doi: 10.1002/jcc.25859 (PMC6767508; doi:10.1002/jcc.25859)
Supplement: Supplementary file 1 — Table S1: Hydrogen bond (H‐bond) energies in α‐helices for AH (α‐helical structure) model, ST (single turn) model, and MH (minimal H‐bond) model by the MTA method and those by the MM computation with AMBER ff99SB force field parameters. The stabilization energies, ΔE SE total, defined by eq. 4 are also listed. The energy minimized α‐helix structures were constructed following the procedure mentioned in the Method section. Table S2: Hirshfeld atomic charges of the carbonyl group (C=O) of i‐th residue and those of the amide group (NH) of (i+4)‐th residue in ST, APN, APC and MH models, for G 0, G 1 and G 2 fragments (see Fig. 2), respectively. The differences are also shown. The optimized six α‐helix structures were constructed following the procedure mentioned in the Method section. Table S3: H‐bond energies in α‐helices for HTN, HTC, APN, and APC models computed by the MTA method with DFT. Table S4: H‐bond energies in α‐helices for AH model in water (ε = 78.3553) with Polarizable Continuum Model (PCM). The α‐helix structures are the same as those in Table S1. Figure S1: Fragment structures and their energies shown in the parentheses (kcal/mol) used in the current MTA method for Ace‐(Ala)3‐Nme: (A) model F 1 (−822.4756), (B) F 2 (−822.4808), (C) F 3 (−822.4831), (D) F 4 (−822.4831), (E) F 12 (−655.0602), (F) F 13 (−655.0649), (G) F 14 (−655.0679), (H) F 23 (−655.0669), (I) F 24 (−655.0714), (J) F 34 (−655.0670), (K) F 123 (−487.6508), (L) F 124 (−487.6559), (M) F 134 (−487.6568), (N) F 234 (−487.6562), (O) F 1234 (−320.2462). Those energies were used in eq. 1 to compute the total MTA energy. The thin green lines are the original Ace‐(Ala)3‐Nme. Figure S2: Electron density change upon H‐bond formation Δρ MTA given by eq. 3 in AH models for structures of (A) 8–2, (B) 8–3, (C) 8–4, (D) 8–5, and (E) 8–6. Yellow surface is the contour surface at −0.001 au, and magenta one is that at 0.001 au. The atoms in MH models are shown by thick stick model and the other atoms in ST m [file JCC-40-2043-s001.docx]

**Supplementary Materials**

Hydrogen Bond Donors and Acceptors are Generally Depolarized

in α-Helices as Revealed by a Molecular Tailoring Approach

Hiroko X. Kondo, Ayumi Kusaka, Colin K. Kitakawa, Jinta Onari,

Shusuke Yamanaka, Haruki Nakamura, and Yu Takano

Table S1

Hydrogen bond (H-bond) energies in α-helices for AH (α-helical structure) model, ST (single turn) model, and MH (minimal H-bond) model by the MTA method and those by the MM computation with AMBER ff99SB force field parameters. The stabilization energies, Δ*E*_SE_^total^, defined by equation (4) are also listed. The energy minimized α-helix structures were constructed following the procedure mentioned in the Method section.

| H-bond ID | Distance^[a]^  (å) | CO-HN angle^[b]^  (degree) | H-bond Energy (kcal/mol) | | | | Δ*E*_SE_^total^ ^[g]^  (kcal/mol) |
| --- | --- | --- | --- | --- | --- | --- | --- |
|  |  |  | AH  model^[c]^ | ST  model^[d]^ | MH  model^[e]^ | MM^[f]^ |  |
| 3-1 | 2.418 | 171.796 | –2.875 | –2.870 | –4.018 | –4.078 | –4.880 |
| 4-1 | 2.363 | 175.977 | –2.980 | –3.296 | –4.509 | –4.425 | –5.361 |
| 4-2 | 2.343 | 167.386 | –2.786 | –2.969 | –4.004 | –3.839 | –4.925 |
| 5-1 | 2.332 | 178.102 | –3.352 | –3.519 | –4.780 | –4.622 | –5.639 |
| 5-2 | 2.268 | 168.123 | –2.911 | –3.420 | –4.533 | –4.296 | –5.458 |
| 5-3 | 2.421 | 164.590 | –2.470 | –2.567 | –3.492 | –3.257 | –4.471 |
| 6-1 | 2.313 | 178.503 | –3.652 | –3.549 | –4.829 | –4.707 | –5.695 |
| 6-2 | 2.199 | 168.542 | –3.362 | –3.713 | –4.891 | –4.631 | –5.827 |
| 6-3 | 2.345 | 163.525 | –2.693 | –3.087 | –4.078 | –3.744 | –5.046 |
| 6-4 | 2.366 | 167.549 | –2.856 | –2.806 | –3.832 | –3.672 | –4.788 |
| 7-1 | 2.292 | 178.062 | –3.765 | –3.585 | –4.880 | –4.792 | –5.752 |
| 7-2 | 2.172 | 168.146 | –3.657 | –3.748 | –4.955 | –4.752 | –5.895 |
| 7-3 | 2.292 | 163.714 | –3.126 | –3.378 | –4.408 | –4.009 | –5.385 |
| 7-4 | 2.293 | 167.155 | –3.020 | –3.291 | –4.394 | –4.154 | –5.336 |
| 7-5 | 2.345 | 168.055 | –3.001 | –2.867 | –3.902 | –3.753 | –4.862 |
| 8-1 | 2.273 | 177.763 | –3.902 | –3.632 | –4.945 | –4.878 | –5.821 |
| 8-2 | 2.147 | 168.060 | –3.789 | –3.781 | –5.004 | –4.851 | –5.948 |
| 8-3 | 2.256 | 163.435 | –3.422 | –3.431 | –4.484 | –4.157 | –5.473 |
| 8-4 | 2.220 | 167.585 | –3.504 | –3.588 | –4.753 | –4.495 | –5.708 |
| 8-5 | 2.263 | 167.404 | –3.187 | –3.350 | –4.472 | –4.264 | –5.422 |
| 8-6 | 2.339 | 167.613 | –3.031 | –2.867 | –3.895 | –3.731 | –4.866 |

1. Distance between O atom of C=O at *i*-th residue and H atom of HN at (*i*+4)-th residue.
2. Angle formed by the two vectors of C=O at *i*-th residue and HN at (*i*+4)-th residue.
3. H-bond energies by AH (original α-helical structure) model with MTA method.
4. H-bond energies by ST (single turn) model with MTA method.
5. H-bond energies by MH (minimal H-bond) model with MTA method.
6. Computed by the MM method with AMBER ff99SB force field parameters.
7. Stabilization energy defined by equation (4).

Table S2:

Hirshfeld atomic charges of the carbonyl group (C=O) of *i*-th residue and those of the amide group (NH) of (*i*+4)-th residue in ST, AP_N_, AP_C_ and MH models, for *G*_0_, *G*_1_ and *G*_2_ fragments (see Figure 2), respectively. The differences are also shown. The optimized six α-helix structures were constructed following the procedure mentioned in the Method section.

A) Hirshfeld charges in *G*_0_, where H-bonds are formed between C=O of *i*-th residue and NH of (*i*+4)-th residue for ST and MH models

| Atoms involved in H-bond | Helix model | Structure | | | | | | |  |
| --- | --- | --- | --- | --- | --- | --- | --- | --- | --- |
|  |  | 8-1 | 8-2 | 8-3 | 8-4 | 8-5 | 8-6 | Average | |
| Carbon of C=O of  *i*-th residue in *G*_0_ | ST model | 0.1793 | 0.1734 | 0.1742 | 0.1740 | 0.1732 | 0.1723 | 0.1744 | |
|  | MH model | 0.1744 | 0.1751 | 0.1766 | 0.1748 | 0.1740 | 0.1730 | 0.1747 | |
|  | Difference^[a]^ | 0.0048 | –0.0017 | –0.0024 | –0.0008 | –0.0008 | –0.0007 | –0.0003 | |
| Oxygen of C=O of  *i*-th residue in *G*_0_ | ST model | –0.2677 | –0.2665 | –0.2700 | –0.2697 | –0.2711 | –0.2743 | –0.2699 | |
|  | MH model | –0.3011 | –0.2911 | –0.2925 | –0.2949 | –0.2969 | –0.2993 | –0.2960 | |
|  | Difference^[a]^ | 0.0333 | 0.0247 | 0.0225 | 0.0252 | 0.0258 | 0.0251 | 0.0261 | |
|  | Distance between O*^i^* and O*^i^*^+1^ (å) | 3.439 | 3.547 | 3.528 | 3.497 | 3.519 | 3.530 | 3.510 | |
| Nitrogen of NH of  (*i*+4)-th residue in *G*_1_ fragment | ST model | –0.1170 | –0.1161 | –0.1148 | –0.1157 | –0.1165 | –0.1165 | -0.1161 | |
|  | MH model | –0.1228 | –0.1231 | –0.1217 | –0.1226 | –0.1228 | –0.1208 | -0.1223 | |
|  | Difference^[a]^ | 0.0058 | 0.0070 | 0.0069 | 0.0069 | 0.0063 | 0.0042 | 0.0062 | |
| Hydrogen of NH of  (*i*+4)-th residue in *G*_1_ fragment | ST model | 0.1070 | 0.1029 | 0.1050 | 0.1041 | 0.1053 | 0.1049 | 0.1049 | |
|  | MH model | 0.1135 | 0.1076 | 0.1116 | 0.1100 | 0.1114 | 0.1141 | 0.1114 | |
|  | Difference^[a]^ | –0.0065 | –0.0047 | –0.0065 | –0.0059 | –0.0061 | –0.0092 | -0.0065 | |
|  | Distance between H*^i^*^+3^ and H*^i^*^+4^ (å) | 2.484 | 2.735 | 2.758 | 2.710 | 2.721 | 2.646 | 2.676 | |

[a] Difference is the value in ST model minus that in MH model.

B) Hirshfeld charges in *G*_0_, where H-bonds are formed between C=O of *i*-th residue and NH of (*i*+4)-th residue for AP_N_ and AP_C_ models

| Atoms involved in H-bond | Helix model | Structure | | | | | | |
| --- | --- | --- | --- | --- | --- | --- | --- | --- |
|  |  | 8-1 | 8-2 | 8-3 | 8-4 | 8-5 | 8-6 | Average |
| Carbon of C=O of  *i*-th residue in *G*_0_ | AP_N_ model | – | 0.1713 | 0.1732 | 0.1702 | 0.1699 | 0.1693 | 0.1708 |
|  | MH model | – | 0.1751 | 0.1766 | 0.1748 | 0.1740 | 0.1730 | 0.1747 |
|  | Difference^[b]^ | – | –0.0038 | –0.0034 | –0.0047 | –0.0041 | –0.0037 | –0.0039 |
| Oxygen of C=O of  *i*-th residue in *G*_0_ | AP_N_ model | – | –0.2859 | –0.2842 | –0.2844 | –0.2875 | –0.2899 | –0.2864 |
|  | MH model | – | –0.2911 | –0.2925 | –0.2949 | –0.2969 | –0.2993 | –0.2949 |
|  | Difference^[b]^ | – | 0.0053 | 0.0083 | 0.0104 | 0.0094 | 0.0094 | 0.0086 |
|  | Distance between O*^i^*^-1^ and O*^i^* (å) | – | 3.439 | 3.547 | 3.528 | 3.497 | 3.519 | 3.506 |
| Nitrogen of NH of  (*i*+4)-th residue in *G*_1_ fragment | AP_C_ model | –0.1224 | –0.1226 | –0.1212 | –0.1219 | –0.1214 | – | –0.1219 |
|  | MH model | –0.1228 | –0.1231 | –0.1217 | –0.1226 | –0.1228 | – | –0.1226 |
|  | Difference^[c]^ | 0.0005 | 0.0005 | 0.0004 | 0.0007 | 0.0014 | – | 0.0007 |
| Hydrogen of NH of  (*i*+4)-th residue in *G*_1_ fragment | AP_C_ model | 0.1104 | 0.1051 | 0.1087 | 0.1075 | 0.1088 | – | 0.1081 |
|  | MH model | 0.1135 | 0.1076 | 0.1116 | 0.1100 | 0.1114 | – | 0.1108 |
|  | Difference^[c]^ | –0.0031 | –0.0024 | –0.0029 | –0.0025 | –0.0026 | – | –0.0027 |
|  | Distance between H*^i^*^+4^ and H*^i^*^+5^ (å) | 2.735 | 2.758 | 2.710 | 2.721 | 2.646 | – | 2.714 |

[b] Difference is the value in AP_N_ model minus that in MH model.

[c] Difference is the value in AP_C_ model minus that in MH model.

C) Hirshfeld charges in *G*_1_ and *G*_2_ fragments, where no H-bonds are formed between C=O of *i*-th residue and NH of (*i*+4)-th residue for ST and MH models

| Atoms involved in H-bond | Helix model | Structure | | | | | | |
| --- | --- | --- | --- | --- | --- | --- | --- | --- |
|  |  | 8-1 | 8-2 | 8-3 | 8-4 | 8-5 | 8-6 | Average |
| Carbon of C=O of  *i*-th residue in *G*_2_ fragment | ST model | 0.1746 | 0.1677 | 0.1694 | 0.1690 | 0.1686 | 0.1685 | 0.1696 |
|  | MH model | 0.1683 | 0.1684 | 0.1711 | 0.1688 | 0.1685 | 0.1685 | 0.1689 |
|  | Difference^[d]^ | 0.0063 | –0.0006 | –0.0016 | 0.0001 | 0.0001 | 0.0000 | 0.0007 |
| Oxygen of C=O of  *i*-th residue in *G*_2_ fragment | ST model | –0.2754 | –0.2795 | –0.2798 | –0.2801 | –0.2807 | –0.2826 | –0.2797 |
|  | MH model | –0.3134 | –0.3089 | –0.3062 | –0.3097 | –0.3104 | –0.3110 | –0.3099 |
|  | Difference^[d]^ | 0.0379 | 0.0294 | 0.0264 | 0.0296 | 0.0298 | 0.0284 | 0.0303 |
| Nitrogen of NH of  (*i*+4)-th residue in *G*_1_ fragment | ST model | –0.1186 | –0.1173 | –0.1174 | –0.1178 | –0.1194 | –0.1208 | –0.1186 |
|  | MH model | –0.1242 | –0.1239 | –0.1240 | –0.1245 | –0.1255 | –0.1249 | –0.1245 |
|  | Difference^[d]^ | 0.0057 | 0.0066 | 0.0066 | 0.0066 | 0.0061 | 0.0041 | 0.0060 |
| Hydrogen of NH of  (*i*+4)-th residue in *G*_1_ fragment | ST model | 0.1187 | 0.1209 | 0.1196 | 0.1192 | 0.1188 | 0.1158 | 0.1188 |
|  | MH model | 0.1348 | 0.1350 | 0.1347 | 0.1343 | 0.1336 | 0.1331 | 0.1342 |
|  | Difference^[d]^ | –0.0161 | –0.0141 | –0.0151 | –0.0151 | –0.0148 | –0.0173 | –0.0154 |

[d] Difference is the value in ST model minus that in MH model.

D) Hirshfeld charges in in *G*_1_ and *G*_2_ fragments, where no H-bonds are formed between C=O of *i*-th residue and NH of (*i*+4)-th residue for AP_N_ and AP_C_ models

| Atoms involved in H-bond | Helix model | Structure | | | | | | |
| --- | --- | --- | --- | --- | --- | --- | --- | --- |
|  |  | 8-1 | 8-2 | 8-3 | 8-4 | 8-5 | 8-6 | Average |
| Carbon of C=O of  *i*-th residue in *G*_2_ fragment | AP_N_ model | – | 0.1653 | 0.1684 | 0.1648 | 0.1650 | 0.1654 | 0.1658 |
|  | MH model | – | 0.1684 | 0.1711 | 0.1688 | 0.1685 | 0.1685 | 0.1691 |
|  | Difference^[e]^ | – | –0.0031 | –0.0027 | –0.0040 | –0.0035 | –0.0031 | –0.0033 |
| Oxygen of C=O of  *i*-th residue in *G*_2_ fragment | AP_N_ model | – | –0.3016 | –0.2957 | –0.2971 | –0.2990 | –0.2996 | –0.2986 |
|  | MH model | – | –0.3089 | –0.3062 | –0.3097 | –0.3104 | –0.3110 | –0.3093 |
|  | Difference^[e]^ | – | 0.0073 | 0.0105 | 0.0126 | 0.0115 | 0.0114 | 0.0107 |
| Nitrogen of NH of  (*i*+4)-th residue in *G*_1_ fragment | AP_C_ model | –0.1240 | –0.1236 | –0.1236 | –0.1238 | –0.1242 | – | –0.1239 |
|  | MH model | –0.1242 | –0.1239 | –0.1240 | –0.1245 | –0.1255 | – | –0.1244 |
|  | Difference^[f]^ | 0.0002 | 0.0003 | 0.0004 | 0.0006 | 0.0014 | – | 0.0006 |
| Hydrogen of NH of  (*i*+4)-th residue in *G*_1_ fragment | AP_C_ model | 0.1291 | 0.1295 | 0.1290 | 0.1288 | 0.1284 | – | 0.1289 |
|  | MH model | 0.1348 | 0.1350 | 0.1347 | 0.1343 | 0.1336 | – | 0.1345 |
|  | Difference^[f]^ | –0.0057 | –0.0055 | –0.0056 | –0.0055 | –0.0052 | – | –0.0055 |

[e] Difference is the value in AP_N_ model minus that in MH model.

[f] Difference is the value in AP_C_ model minus that in MH model.

Table S3: H-bond energies in α-helices for HT_N_, HT_C_, AP_N_, and AP_C_ models computed by the MTA method with DFT.

| α-helix  Structure | H-bond Energy (kcal/mol) | | | |
| --- | --- | --- | --- | --- |
|  | HT_N_  model^[a]^ | HT_C_  model^[b]^ | AP_N_  model^[c]^ | AP_C_  model^[d]^ |
| 8-1 | –4.418 | –3.993 | – | –4.516 |
| 8-2 | –4.526 | –4.152 | –4.793 | –4.495 |
| 8-3 | –4.044 | –3.785 | –4.188 | –4.006 |
| 8-4 | –4.273 | –3.959 | –4.408 | –4.273 |
| 8-5 | –4.001 | –3.714 | –4.165 | –4.043 |
| 8-6 | –3.432 | –3.227 | –3.610 | – |

1. N-terminal Half-Turn model shown in Figure S5 (A).
2. HT_C_ (C-terminal Half-Turn) model shown in Figure S5 (B).
3. AP_N_ (Additional N-terminal Peptide) model shown in Figure S5 (C). Because the AP_N_ model was built from Ace-(Ala)_8_-Nme, there was no model for 8-1.
4. AP_C_ (Additional C-terminal Peptide) model shown in Figure S5 (D). Because the AP_C_ model was built from Ace-(Ala)_8_-Nme, there was no model for 8-6.

Table S4: H-bond energies in α-helices for AH model in water (*ε* = 78.3553) with Polarizable Continuum Model (PCM). The α-helix structures are the same as those in Table S1.

| α-helix Structure | H-bond energy (kcal/mol) |
| --- | --- |
| 3-1 | -2.096 |
| 4-1 | -2.435 |
| 4-2 | -1.835 |
| 5-1 | -2.713 |
| 5-2 | -2.352 |
| 5-3 | -1.507 |
| 6-1 | -2.836 |
| 6-2 | -2.717 |
| 6-3 | -2.012 |
| 6-4 | -1.879 |
| 7-1 | -2.791 |
| 7-2 | -2.779 |
| 7-3 | -2.253 |
| 7-4 | -2.315 |
| 7-5 | -1.905 |
| 8-1 | -2.932 |
| 8-2 | -2.902 |
| 8-3 | -2.436 |
| 8-4 | -2.677 |
| 8-5 | -2.531 |
| 8-6 | -1.970 |


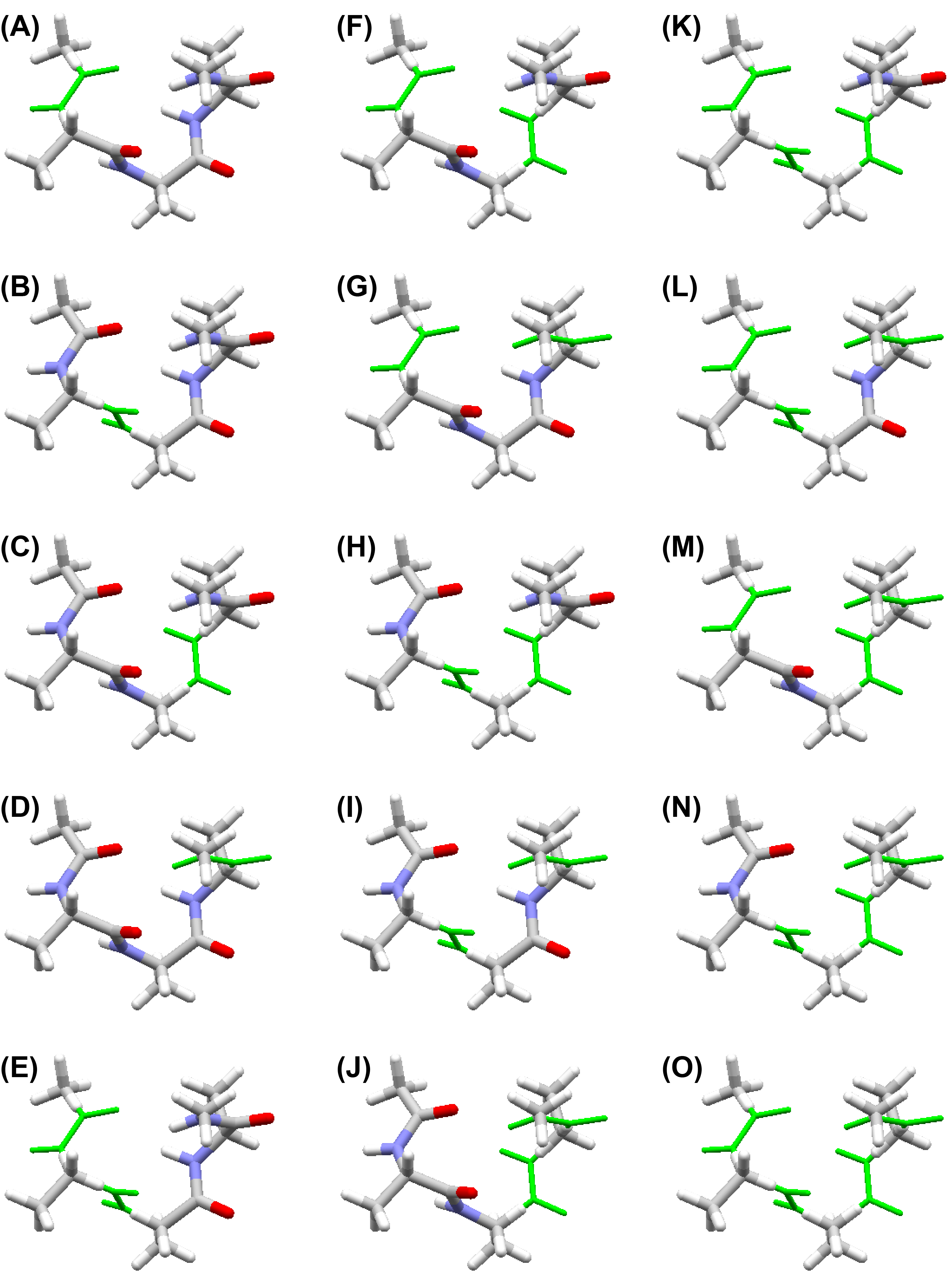


Figure S1: Fragment structures and their energies shown in the parentheses (kcal/mol) used in the current MTA method for Ace-(Ala)_3_-Nme: (A) model *F*_1_ (–822.4756), (B) *F*_2_ (–822.4808), (C) *F*_3_ (–822.4831), (D) *F*_4_ (–822.4831), (E) *F*_12_ (–655.0602), (F) *F*_13_ (–655.0649), (G) *F*_14_ (–655.0679), (H) *F*_23_ (–655.0669), (I) *F*_24_ (–655.0714), (J) *F*_34_ (–655.0670), (K) *F*_123_ (–487.6508), (L) *F*_124_ (–487.6559), (M) *F*_134_ (–487.6568), (N) *F*_234_ (–487.6562), (O) *F*_1234_ (–320.2462)*.* Those energies were used in equation (1) to compute the total MTA energy. The thin green lines are the original Ace-(Ala)_3_-Nme.


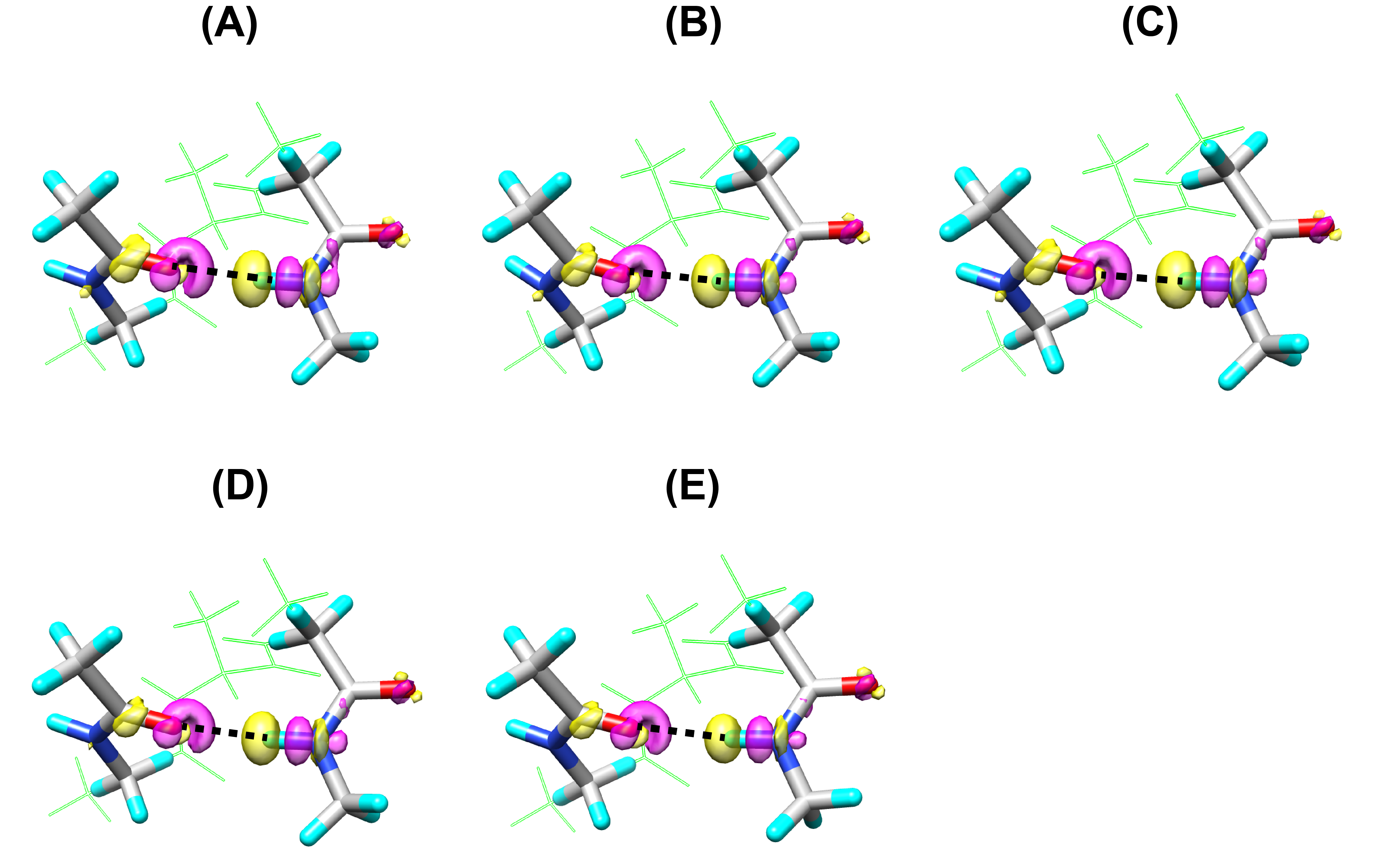
Figure S2: Electron density change upon H-bond formation Δ*ρ*_MTA_ given by equation (3) in AH models for structures of (A) 8-2, (B) 8-3, (C) 8-4, (D) 8-5, and (E) 8-6. Yellow surface is the contour surface at –0.001 au, and magenta one is that at 0.001 au. The atoms in MH models are shown by thick stick model and the other atoms in ST models are shown by thin green lines. Each black dotted line is the H-bond between the oxygen atom of C=O group at *i*-th residue and the hydrogen atom of N-H group at (*i*+4)-th residue.


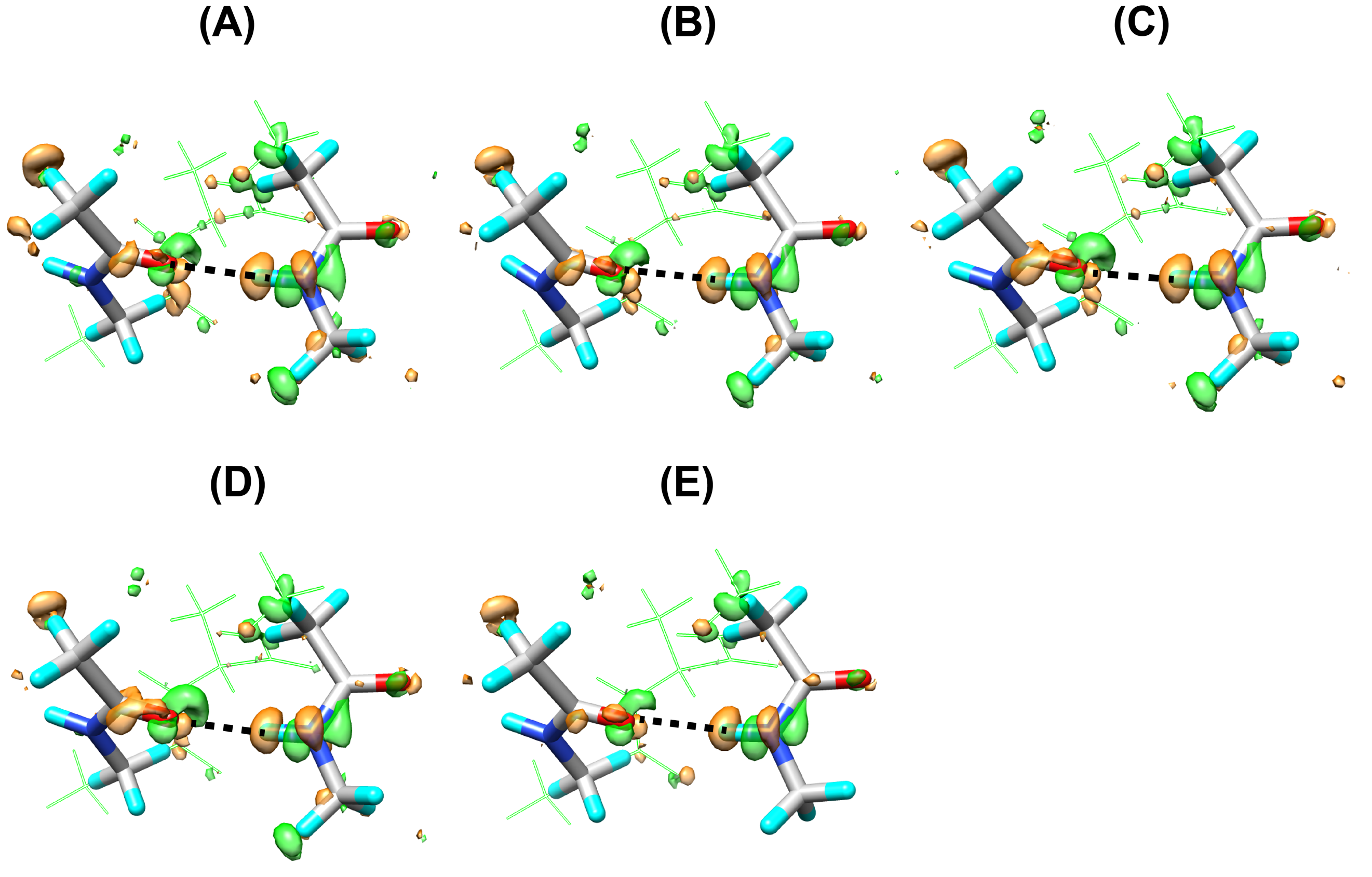
Figure S3: Difference in the electron density change upon H-bond formation between AH and MH models ΔΔ*ρ*_MTA_^AH−MH^ by equation (7) for structures of (A) 8-2, (B) 8-3, (C) 8-4, (D) 8-5, and (E) 8-6. Green surfaces are the contour surfaces at -0.0002 au, and orange ones are those at 0.0002 au. The atoms in MH models are shown by thick sticks, and other atoms in ST models are by thin green lines. The black dotted lines are the H-bonds between the oxygen atoms of C=O groups at *i*-th residues and the hydrogen atoms of N-H groups at (*i*+4)-th residues.


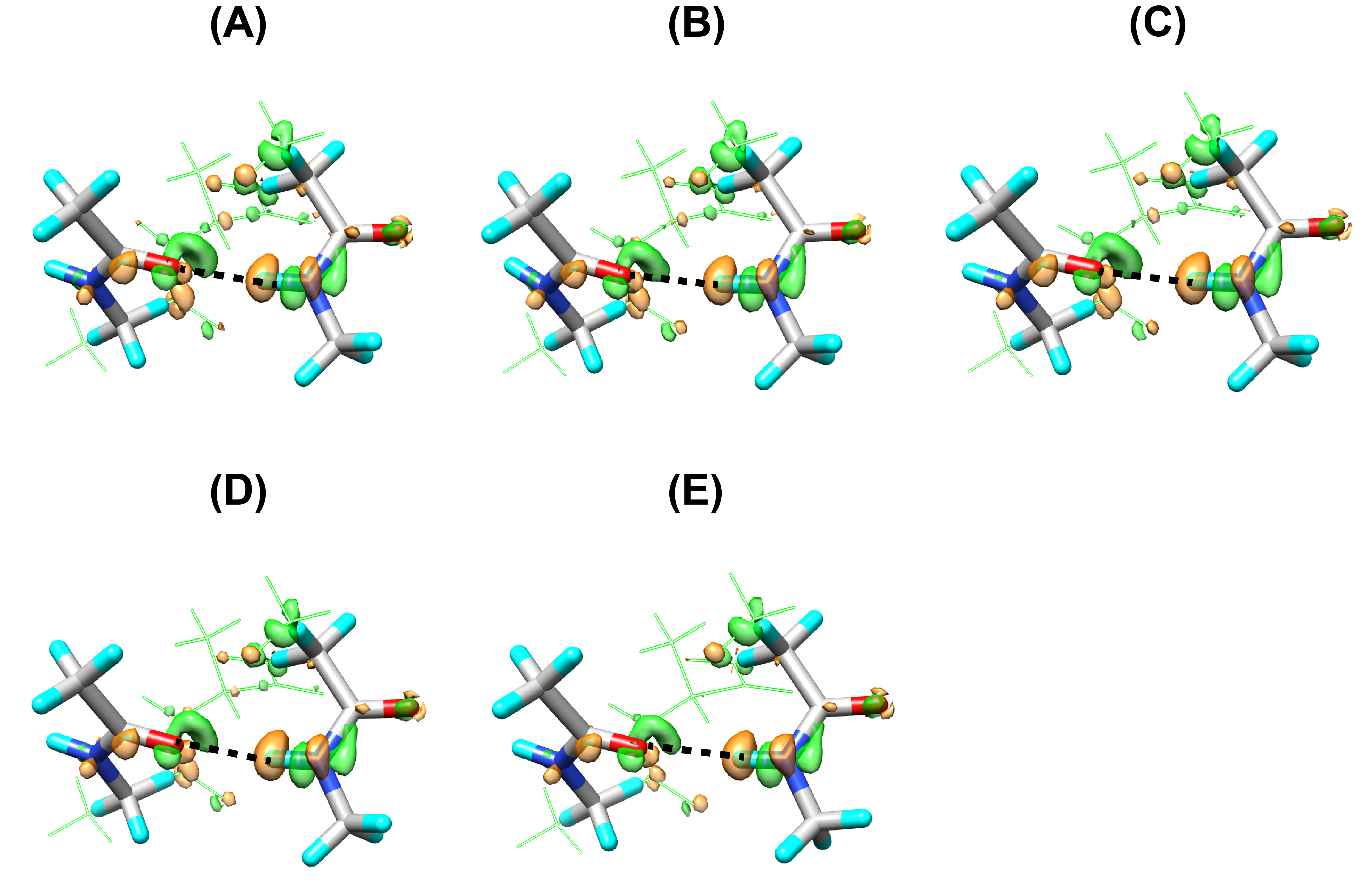
Figure S4: Difference in the electron density change upon H-bond formation between ST and MH models ΔΔ*ρ*_MTA_^ST–MH^ by eq. [7] for structures of (A) 8-2, (B) 8-3, (C) 8-4, (D) 8-5, and (E) 8-6. Green surfaces are the contour surfaces at -0.0002 au, and orange ones are those at 0.0002 au. The atoms in MH models are shown by thick sticks, and other atoms in ST models are by thin green lines. The black dotted lines are the H-bonds between the oxygen atoms of C=O groups at *i*-th residues and the hydrogen atoms of N-H groups at (*i*+4)-th residues.


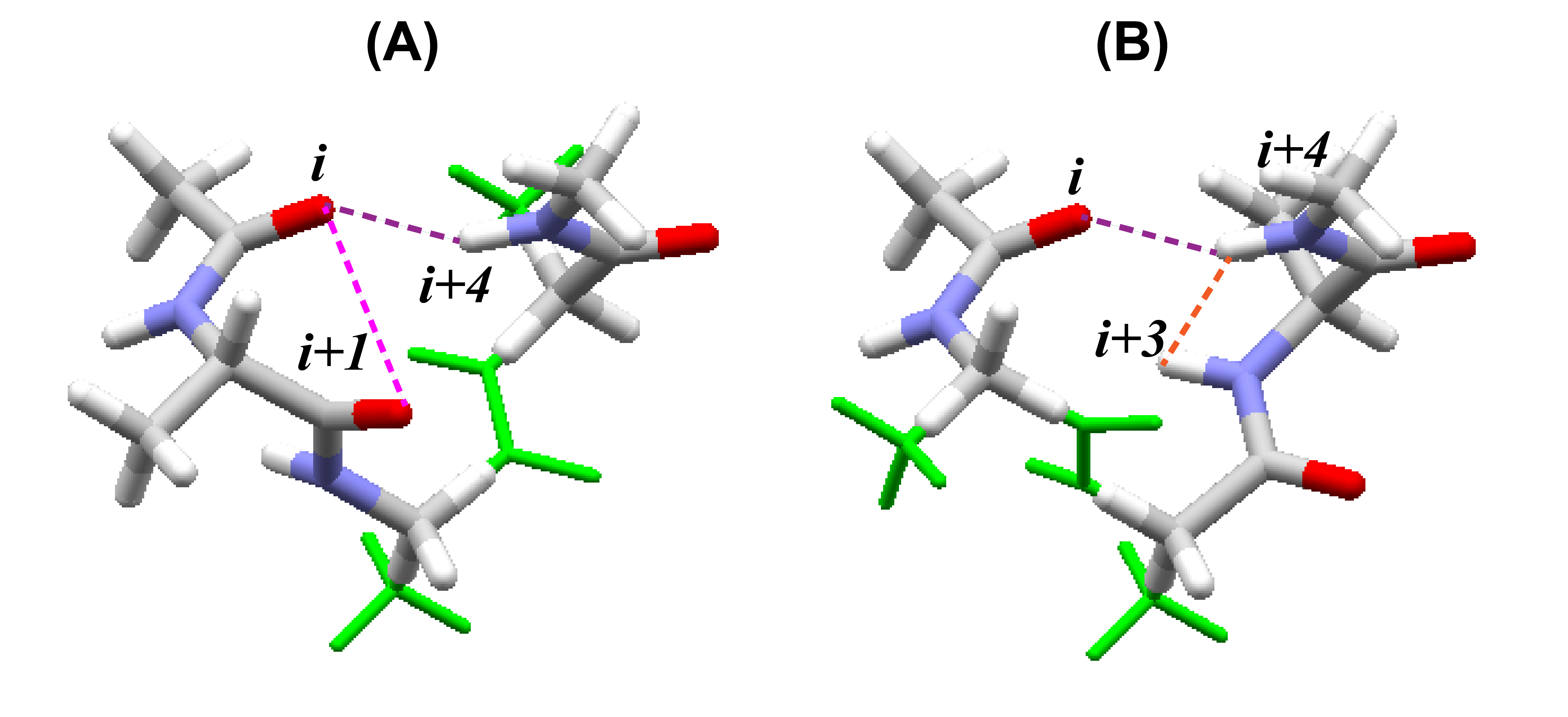


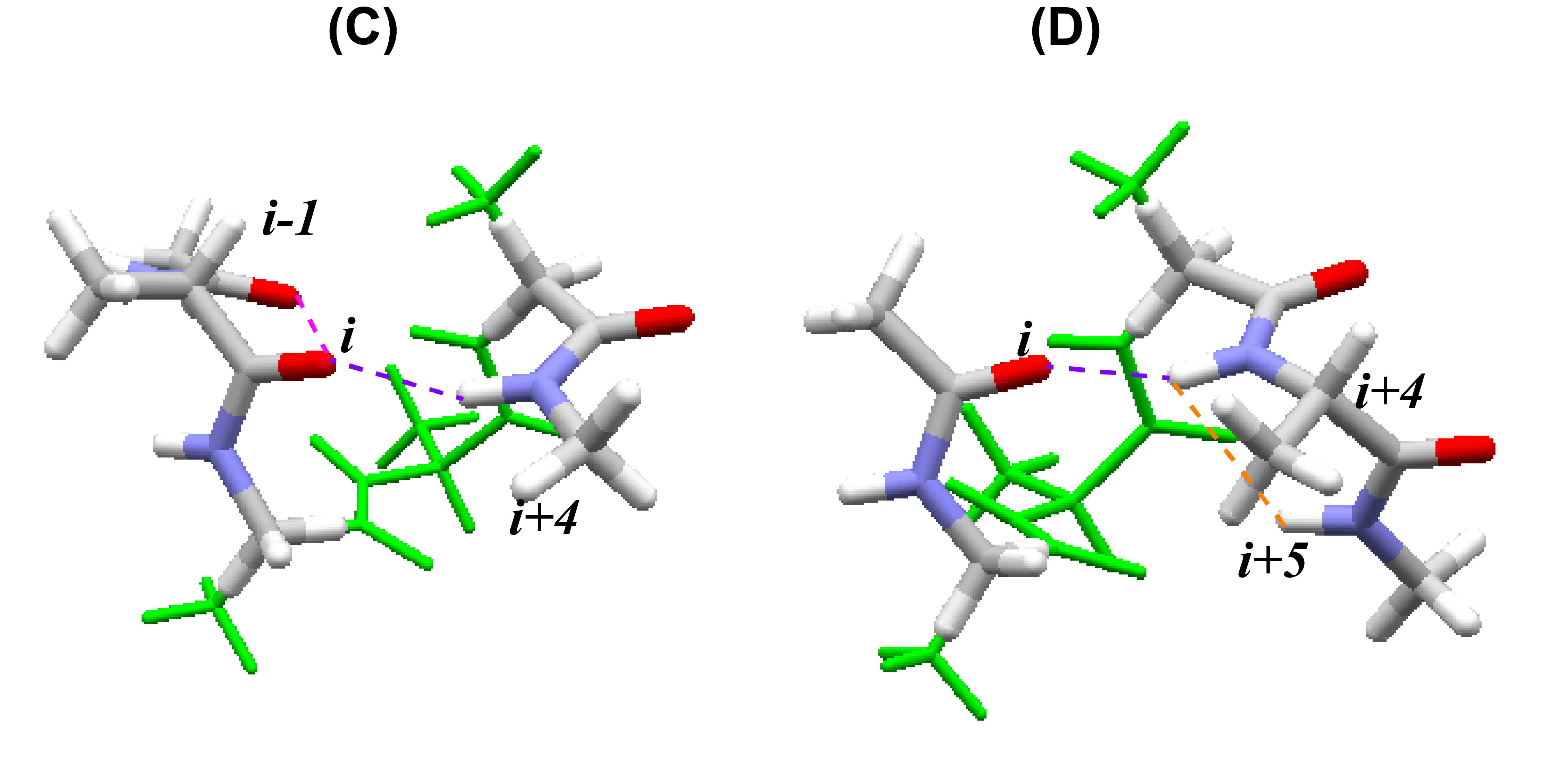


Figure S5: (A) HT_N_ (N-terminal Half-Turn) model, and (B) HT_C_ (C-terminal Half-Turn) model. The N-terminus of HT_N_ model and the C-terminus of HT_C_ model are both Ace-Ala-Nme, instead of the minimal Ace-Nme groups. Thus, HT_N_ model has an interaction between the successive C=O groups at *i*-th and (*i*+1)-th residues indicated by a magenta dotted line in (A), and HT_C_ model has another interaction between the successive NH groups at (*i*+3)-th and (*i*+4)-th residues indicated by an orange dotted line in (B). The target H-bonds between C=O groups at *i*-th residue and NH groups at (*i*+4)-th residues are indicated by purple dotted lines. The thin green lines are the original ST model, Ace-(Ala)_3_-Nme. (C) AP_N_ (N-terminal Additional-Peptide) model, and (D) AP_C_ (C-terminal Additional-Peptide) model. Ace-Ala group and Ala-Nme group are added at the N-terminus of AP_N_ model and the C-terminus of AP_C_ model, respectively. Thus, AP_N_ model has an interaction between the successive C=O groups at (*i*–1)-th and *i*-th residues indicated by a magenta dotted line in (C), and AP_C_ model has another interaction between the successive NH groups at (*i*+4)-th and (*i*+5)-th residues indicated by an orange dotted line in (D). The target H-bonds between C=O groups at *i*-th residue and NH groups at (*i*+4)-th residues are indicated by purple dotted lines. The thin green lines are the original ST model, Ace-(Ala)_3_-Nme.


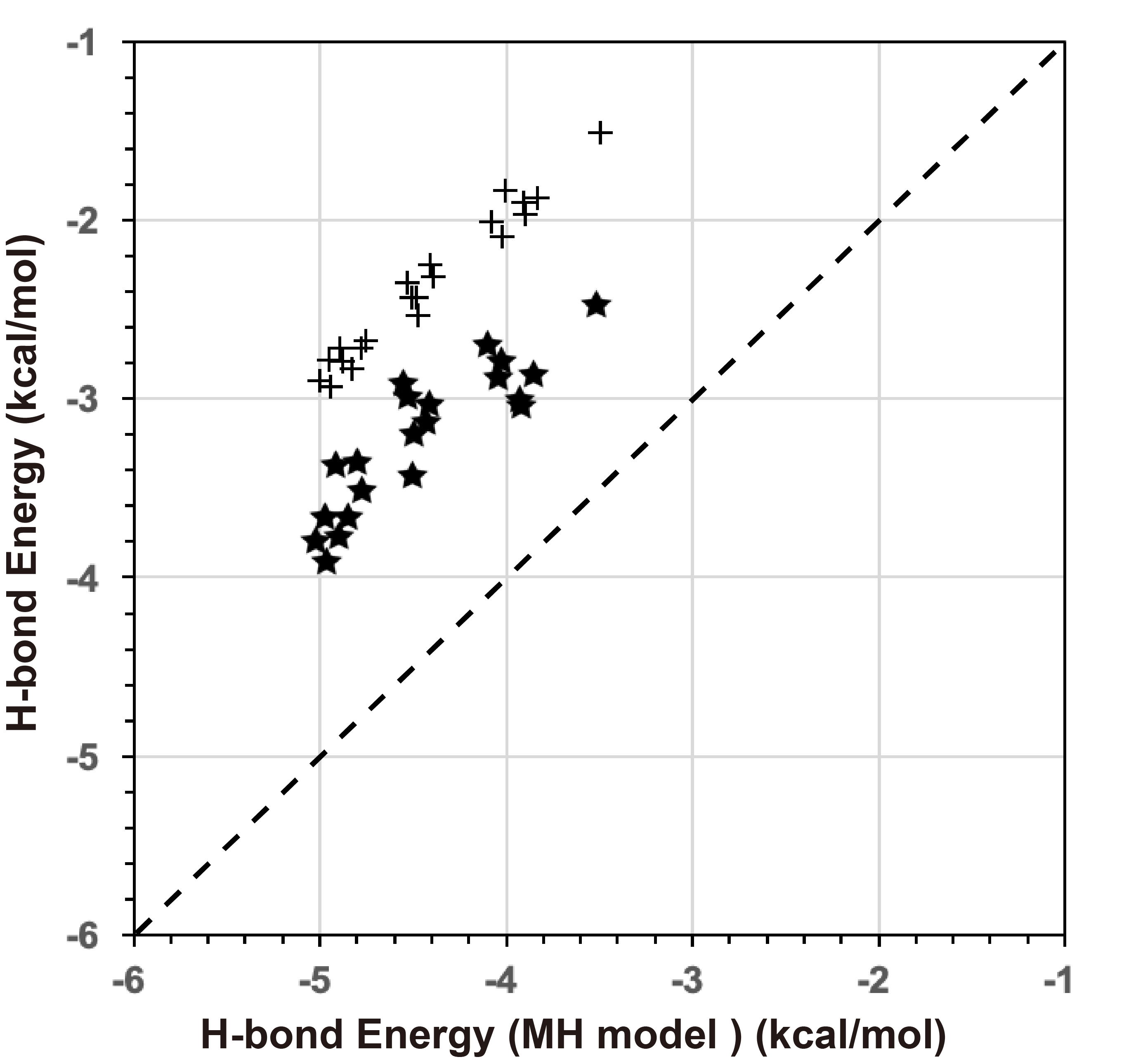


Figure S6: Correlation of the H-bond energies of AH model in water (ε = 78.3553) with Polarizable Continuum Model (PCM) (cross symbol) against those by MH model. For comparison, the correlation of the H-bond energies of AH model *in vacuo* in Figure 4 is also shown by filled star. The dashed line shows a guide where the longitudinal axis values have the same H-bond energies by MH models.
